# “Cheese: Technology, Compositional, Physical and Biofunctional Properties:” A Special Issue

**DOI:** 10.3390/foods8100512

**Published:** 2019-10-18

**Authors:** Golfo Moatsou

**Affiliations:** Laboratory of Dairy Research, Department of Food Science and Human Nutrition, Agricultural University of Athens, Iera Odos 75, 11855 Athens, Greece; mg@aua.gr

**Keywords:** cheese, special issue

## Abstract

The objective of the present editorial to critical synopsize articles that make up the Special Issue “Cheese: Technology, Compositional, Physical and Biofunctional Properties.” The published research papers are multidisciplinary studies which refer to some of the most important sub-topics of Cheese Science and Technology. They present the results of experimental studies and analyses that can be further exploited by academia and cheese producers.

## 1. Introduction

Cheese is a very popular food produced worldwide from the milk of ruminants using a combination of physical treatments. Key milk components for the transformation of milk into curd are casein and calcium. The majority of cheese varieties are based on the curd of modified casein micelles that result from the enzymatic rennet clotting of milk in the presence of calcium ions. The remarkable ability of the spontaneous syneresis of rennet-induced curds can be adjusted to the desired level by biological acidification, cutting, stirring, heating, pressing and salting in order to achieve the desired level of water removal in the form of whey. The mode of curdling (acid or rennet coagulation), the conditions, and the combinations of curd treatments result in numerous cheese varieties with different appearances, textures, flavors and shelf lives. Moreover, most of them are kept under specific temperature and humidity conditions to ripen for a short or a considerable amount of time. The classification of cheese varieties is not unambiguous and can be based on various criteria. For example, cheeses can be classified according to their moisture content related to yield and shelf life or according to specific features related to the treatments applied during cheesemaking and ripening. During ripening, the main solid constituents of young cheese—fat and caseins—undergo changes that increase the concentration of small size compounds in cheese—such as peptides, amino acids, small volatile molecules—control moisture loss and configure textural properties. In particular, the compounds of mature cheese flavor result from complicated biochemical pathways that take place during cheese ripening or even storage. 

Numerus research papers and several books (e.g., recent [1,2,3,4,5,6]) for cheese have been published from various research groups worldwide that have compiled scientific findings and technological applications. 

## 2. Articles of the Special Issue

Cheese is a very complicated food matrix, and a multidisciplinary approach is therefore necessary for the study or the development of a cheese product. The research papers published in the present Special Issue fulfill this condition. They refer to some of the most important sub-topics of Cheese Science and Technology and present the results of experimental studies and analyses.

The article by Sánchez-Gamboa et al. [7] is a study of the effect of environmental factors, such as the season of the year and variable artisanal practices, on the features and the evolution of the ripening of a raw milk semi-hard/hard Mexican cheese. The absence of a heat treatment of cheese milk indicated the significant effect of the season of the year on the microbiota profile of cheese. The same was true for the microbiological and compositional features of cheese when non-standardized manufacturing practices were applied in various manufacturing places, although most textural parameters were not significantly affected. It was proven that 180–270 days of ripening were necessary for the elimination of coagulase-positive staphylococci, depending on the initial counts. Moreover, after 270 days of ripening, one log cfu/g coliforms still remained in the cheese mass. Among others findings, this study indicates the need for the standardization of artisanal cheese-making conditions, especially when raw cheese milk is utilized.

The color of the rind of smear-ripened cheeses is the objective of the research paper by Sutthiwong et al. [8]. They report that the yellow colonies of *Arthrobacter arilaitensis* (now *Glutamicibacter arilaitensis*) greatly contribute to the pigmentation of this type of rind. In this respect, they performed experiments by means of cheese-based (curd) solid media to investigate the effects of initial pH, NaCl level, and chemical and biological deacidification. The results and their discussion show the complexity of the phenomena that take place on cheese rind. According to the findings, NaCl inhibits color development only under low initial pH conditions, e.g., at pH 5.8, whereas the coloring capacity of bacterial species was differentiated according to the deacidification yeast species. They concluded that the control of pH change during cheese ripening using particular yeast strains is necessary for the development of a typical smear color acceptable to consumers.

In their article, Castada et al. [9] present the results of a study on the flavor of Swiss cheese. They analyzed the volatile compounds of typical Swiss cheese samples made in different cheese factories with the aim to investigate the variation of flavor compounds and to correlate their concentration profiles with sensory analysis and consumer liking. There were correlations among volatile compounds, and the most discriminating of them were classified into five functional groups. Specific sensory attributes were correlated with sub-sets of volatile compounds indicating the flavor complexity of this cheese type and the relationship between volatile compounds and particular organoleptic characters. Based on their findings, the authors suggest that the use of the estimated correlations can be a tool for the improvement of the consistency of the flavor of Swiss cheese produced in various cheese factories. 

The objective of the research paper of Cankurt [10] is to investigate an alternative treatment for the reduction of salt content of a cheese-in-brine. In this respect, various stabilizers were added to the keeping brine of a white cheese with an 8% salt content that was much lower than the 12% salt content of the control brines. The findings showed that the keeping of cheese in loose or stable brine gels with a lower salt content did not downgrade the textural and sensory properties of cheese, whereas it favored the decrease of pH. The kind of stabilizer affected the textural properties in a different manner, with guar gum exhibiting the least desirable effect. The author suggest that the gel-type structure of the brines did not favor the exchange phenomena between cheese and keeping medium.

The development of reduced-fat and reduced-sodium, semi-hard sheep milk cheese is presented in the manuscript of Moatsou et al. [11]. The partial substitution of NaCl with KCl and the incorporation of denatured whey proteins were the experimental treatments. The latter was carried out through the addition of commercial microparticulated whey proteins (MWP) and through the in-situ partial denaturation by means of the high pasteurization of cheese milk. The characteristics of cheeses and the evolution of ripening were assessed using physicochemical analyses, proteolysis indices, texture profile analysis, and organoleptic evaluation. According to the results, all the interventions resulted in reduced-fat cheeses with moisture-on-fat-substances (MNFS) similar to their full-fat counterparts. Furthermore, the in-situ partial denaturation of WP was more effective inducing significant increases of moisture, level of acidification, and total organoleptic score. Bitterness or metallic taste was not detected, whereas cheese features were marginally or not impaired. The authors suggest that the combination of the applied cheesemaking technology with the in-situ whey protein denaturation and the salting in NaCl/KCl brine is adequate for the improvement of the nutritional characteristics of reduced-fat sheep milk cheese.

The effect of salting conditions—dry salting for 24 h or brine salting with various NaCl concentrations at different temperatures for different time intervals—on Halloumi cheese is investigated in the article of Kaminarides et al. [12]. Cheese analyses showed that the mineral content and profile of cheese were affected by the way of salting more intensively than their textural properties. Brine salting substantially decreased the calcium and potassium content of cheeses, thus impairing the nutritional value of brine-salted cheeses. The same was true for the increase of NaCl concentration and of brining temperature. The textural features of dry-salted cheeses were more advantageous than those of their counterparts salted in brines with more than 7% brine. The authors conclude that dry-salting is the most appropriate procedure for Halloumi cheese, whereas the use of brine at 4 °C seems more adequate than that at 20 °C.

## 3. Conclusions

The small collection of articles of this Special Issue are representative of the multidisciplinary character of the research on cheese, and their results can be further exploited by both academia and cheese producers.
